# Quantum nanophotonics with group IV defects in diamond

**DOI:** 10.1038/s41467-019-13332-w

**Published:** 2019-12-09

**Authors:** Carlo Bradac, Weibo Gao, Jacopo Forneris, Matthew E. Trusheim, Igor Aharonovich

**Affiliations:** 1 School of Mathematical and Physical Sciences, Faculty of Science, University of Technology, Sydney, NSW 2007 Australia; 2 Division of Physics and Applied Physics, School of Physical and Mathematical Sciences, Nanyang Technological University, Singapore, 637371 Singapore; 3 Istituto Nazionale di Fisica Nucleare (INFN) and Physics Department, Università degli Studi di Torino, Torino, 10125 Italy; 4 Department of Electrical Engineering and Computer Science, Massachusetts Institute of Technology, Cambridge, MA 02139 USA

**Keywords:** Nanoscience and technology, Optics and photonics

## Abstract

Diamond photonics is an ever-growing field of research driven by the prospects of harnessing diamond and its colour centres as suitable hardware for solid-state quantum applications. The last two decades have seen the field shaped by the nitrogen-vacancy (NV) centre with both breakthrough fundamental physics demonstrations and practical realizations. Recently however, an entire suite of other diamond defects has emerged—group IV colour centres—namely the Si-, Ge-, Sn- and Pb-vacancies. In this perspective, we highlight the leading techniques for engineering and characterizing these diamond defects, discuss the current state-of-the-art group IV-based devices and provide an outlook of the future directions the field is taking towards the realisation of solid-state quantum photonics with diamond.

## Abstract (web summary)

Diamond colour centres have applications in quantum sensing, quantum communication and other important technologies. Bradac et al. survey the progress made in using group IV defect centres, which are anticipated to have practical advantages over the more commonly-used nitrogen vacancy centres.

## Introduction

The field of diamond photonics is marching into its third decade—its birth arguably marked by the 1997 discovery of room temperature optically detected magnetic resonance from a single diamond nitrogen-vacancy (NV) centre.^1^ The unique ability of the NV’s spin to be initialized, manipulated and optically read out at room temperature gave substance to the aspiration of realizing solid-state quantum bits operating in ambient conditions.^2,3^ Tremendous efforts followed, driven by the goal to engineer high-quality NV centres with long spin coherence times, and ameliorate the fabrication of diamond nanostructures for efficient light extraction.^4–7^ The remarkable progress made in pursuit of this endeavour resulted in landmark realizations both in fundamental and applied science including on-demand entanglement,^3^ nanoscale nuclear magnetic resonance^8,9^ and quantum memories.^10^

Nonetheless, these realizations revealed how advanced quantum applications require specific characteristics for the single-photon emitter candidate. For quantum communication, it is desirable for the emitter to have high quantum efficiency, high Debye–Waller factor, short lifetime and negligible spectral diffusion. Additionally, for quantum sensing and quantum computing the source should have an addressable spin state to encode the information, which can be initialized, manipulated and read out, and with a coherence time that is a few-order-of-magnitude longer than the time required to perform a fundamental operation on the state itself. Consequently, for some applications that require better photon throughput, for example, quantum repeaters, the NV centre is not ideal. Its long fluorescent lifetime (~11 ns) and weak emission into the zero-phonon line (ZPL) (only ~4% at room temperature) put an upper bound to the maximum photon rates achievable when employing NV centres in basic quantum photonic devices. While significant progress has been made towards improving photon extraction—for instance, via the use of solid immersion lenses, diamond antennas or optical resonators—having photon emitters with better-suited properties is a more straightforward path to performance gains.

In the recent past, other colour centres in diamond have thus been explored. Initial experiments revealed a diverse spread of narrowband emitters, spanning over the visible and near-infrared spectral range.^11^ The silicon-vacancy (SiV^–^) colour centre was the first of the group IV atoms to be investigated, as it was a known colour centre in diamond from the early 1980s^12^—yet only unambiguously identified as a silicon-related defect with its current electronic level structure in the 1990s.^13,14^ Unfortunately, the first studies on single SiV^–^ defects created by ion implantation gave mixed results.^15^ Although the centre possesses a sharp ZPL at 738 nm (responsible for >70% of the total emission) with only weak vibronic sidebands at room temperature, and a short photoluminescence lifetime of ~1 ns, it also displayed low single-photon emission rates (a few kcounts/s) and low radiative quantum yield (~0.05).

**Fig. 1:**
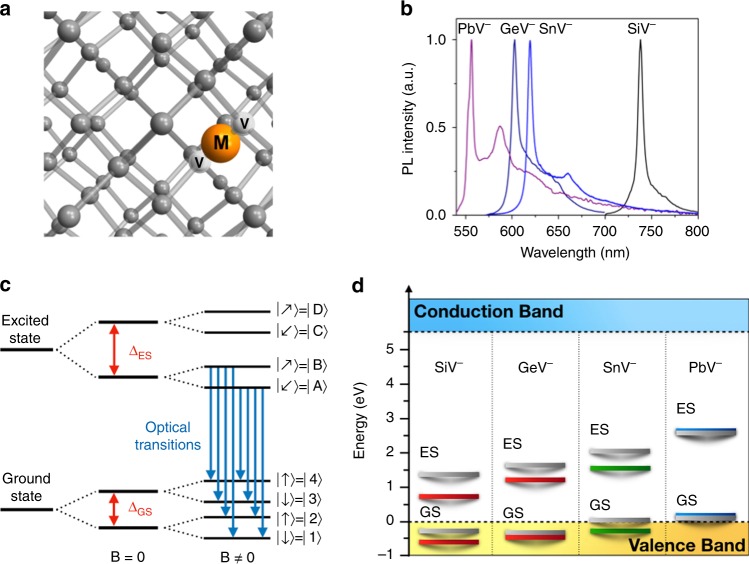
Photoluminescence of group IV centres in diamond. **a** Atomic structure of group IV colour centres in the *D*_*3d*_ symmetry, split-vacancy configuration. The group IV element (M, orange) lies between two nearest-neighbours missing carbon atoms (V, white). **b** Typical room temperature photoluminescence (PL) spectrum of SiV^–^, GeV^–^, SnV^–^ and PbV^–^ centres. **c** Energy structure of impurity-divacancy centres within zero and non-zero external magnetic field *B*. **d** Predicted energies of the ground (GS) and excited (ES) states of the group IV colour centres with respect to the diamond valence band maximum (i.e. the location of the valence band at the Brillouin-zone centre). Levels in grey, red, green and blue refer to values calculated in refs. ^29,32,34,40^.

In 2011, the centre was revisited after a study from Neu et al.^16^ showed ultra-bright emission (~4.8 Mcounts/s, at saturation) from single diamond SiV^–^ defects grown via microwave-plasma-assisted chemical vapour deposition (CVD) on an iridium substrate. This was the first time single SiV^–^ defects could be isolated using growth and displayed high count rates. The discovery reinvigorated the interest for this defect, which resulted into better understanding of its level structure,^17,18^ photophysical properties^19,20^, and spin coherence times, as well as driving the design of schemes for initialization, readout, and coherent preparation of the centre.^21,22^ Remarkably, the SiV^–^ centre shows nearly lifetime-broadened optical emission, in both nanodiamonds^23,24^ and bulk crystals.^25^ This derives from its inversion symmetry (group *D*_3d_, with the silicon atom in a split-vacancy configuration) protecting the optical transitions from local electric field fluctuations,^26^ which in turn allows for the existence—and potentially fabrication—of multiple, intrinsically identical emitters in high-quality bulk diamond.^27^ However, one of the main drawbacks of the SiV^–^ is its aforementioned, intrinsically low quantum efficiency—alongside sub-microsecond spin coherence time even at cryogenic temperatures.^21,28^ This prompted researchers around the world to explore—naturally—other group IV elements foreseeing that they will form colour centres with the same symmetry, and anticipating some of these might have a combination of desired properties with no, or limited, shortcomings. In 2015, the germanium-vacancy (GeV) centre was identified,^29–31^ followed by the tin-vacancy (SnV)^32,33^ and the lead-vacancy (PbV) centres.^34,35^ These colour centres exhibit similar optical properties to the well-studied SiV (e.g. in terms of linewidth and Debye–Waller factor), while potentially offering unique attributes. For instance, due to the higher atomic mass, the ground-state splitting is envisioned to be larger, resulting in potentially less spin mixing and higher qubit operation temperatures. Having non-silicon-related colour centres also opens interesting perspectives for controlled doping, since silicon is known to be a common impurity in CVD diamond chambers. The main properties of the group IV defects in diamond are summarized in Table 1 and their structure and level diagram are shown in Fig. 1. In this perspective, we focus specifically on the negatively charged group IV diamond colour centres (M-V^–^) for they are the most commonly observed and studied. Apart from the SiV^0^, the neutrally charged counterparts (M-V^0^) have not been yet observed. We nonetheless discuss the neutrally charged state of the centres in a dedicated section of the paper (cf. ‘Charge-state control’).

**Table 1:** Photophysical properties of the group IV defects.

	ZPL	FHWM at RT	Excited state Lifetime	Ground-state splitting	Spin–lattice relaxation *T*_1_	Transverse relaxation time *T*_2_
SiV^–^	738 nm	0.7–5 nm [ref. ^16^]	1.0–2.4 ns [ref. ^16^]	~50 GHz [ref. ^36^]	>1 s, at 100 mK [ref. ^36^]	*T*_2_ ~13 ms, at 100 mK [ref. ^36^]
GeV^–^	602 nm	~5 nm [ref. ^29^]	1.4–5.5 ns [ref. ^29^]	~170 GHz [ref. ^37^]	25 μs, at 2 K [ref. ^37^]	*T*_2_* ~20 ns [ref. ^37^]
SnV^–^	620 nm	~6 nm [ref. ^32^]	~6 ns [ref. ^33^]	∼850 GHz [ref. ^32^]	~60 μs [ref. ^38^]	Unknown
PbV^–^	520 nm, 552 nm	~7 nm [ref. ^34,35^]	>3 ns [ref. ^34^]	5.7 THz, at 520 nm 4.2 THz, at 552 nm [ref. ^34^]	Unknown	Unknown

For detailed discussion and references, see main text

This perspective highlights the progress made along this journey—the challenges met and the successes achieved while engineering and characterizing new group IV emitters, as well as testing their performance for the realization of scalable quantum photonic devices. We start with presenting the general optical properties of these emitters and the methods developed to engineer them—focusing on ion implantation. We then discuss the spin coherence properties of the SiV^–^ and the GeV^–^ centres, and show the current state of the art for a few representative quantum photonic and quantum plasmonic experiments realized with these emitters. We conclude by giving a broad outlook over the outstanding obstacles, which might drive future directions and advancements in the field. For the broader field of diamond photonics and the photo-physics of the NV centre in diamond, we refer the reader to other excellent reviews.^5–7,39^

## Fabrication strategies

One major aspect, which is currently shaping the field, is the development of methods for the deterministic—ideally scalable—fabrication of group IV defects. These emitters are most commonly produced through either impurity incorporation during synthesis or via targeted ion implantation. For the former, one of the main challenges is demonstrating controlled growth of single emitters using CVD. While for the latter, accurate and precise placement as well as high conversion efficiency of the implanted ion into an optically active colour centre are the desirable features.

### Incorporation

The formation of SiV^–^ centres in diamond is rather simple. Due to the residual silicon, often present in CVD chambers from quartz bell jars or substrates, most of CVD-grown nanodiamond does contain SiV^–^ centres.^16,41^ Likewise, many high-pressure high-temperature (HPHT) nanodiamonds and bulk single crystals can include SiV^–^ centres that are present due to the addition of silicon-containing precursors.^42^ Similarly, the incorporation of GeV^–^ centres in diamond has been demonstrated by utilizing suitable precursors or substrates both during HPHT and CVD synthesis, while SnV^–^ has only been synthesized via HPHT synthesis, so far.^43–48^ The effectiveness of the synthesis depends on the solubility of the specific element, and thus decreases as the atomic radius increases. It should be noted that the synthesized emitters generally exhibit better optical properties, for example, higher photostability and narrower emission bandwidth, than those of centres fabricated by ion implantation—mainly owing to the higher crystalline quality of the former.^24,37^ However, this approach has a significant drawback in the context of device fabrication—the synthesis process only offers statistical control on the number of emitters and does not allow for precise positioning. For this reason, incorporation during synthesis is arguably best suited for hybrid approaches involving the fabrication of emitters in, for instance, individual diamond nanoparticles, which are subsequently integrated with external photonic structures by pick-and-place techniques.^49^

### Ion implantation

This approach stems from an enticing perspective: to individually control the number and position of the colour centres in the diamond material—ideally forming arbitrary arrays of quantum emitters. Ion implantation involves bombarding the host material with selected ions and subsequently performing an annealing step at high temperatures (usually >800 °C). The annealing process heals the damage produced by the ion bombardment to the crystal and provides energy for the mobilization of self-interstitials and isolated vacancies, thus favouring the formation of M-V complexes. Ion implantation is a technologically mature method, which inherits a large body of knowledge from silicon-based electronic device manufacturing. Its exploitation for quantum device fabrication is still limited as it currently lacks reliable, sub-50 nm positioning accuracy, single-ion delivery, and formation yields close to unity (i.e. ratio between optically active emitters and number of implanted ions).^50^

### Spatial accuracy

The deterministic positioning of colour centres in diamond via implantation is intrinsically limited by lateral straggling, that is, scattering of the ions as they travel through the target material. Notably, the relatively high mass of group IV ions lessens the effects of straggling in diamond enabling sub-30 nm implantation accuracy with energies that, for instance for Si ions, can be of the order of 200 keV. Higher placement accuracies (<10 nm) can be achieved at lower energies and for heavier elements (Ge–Pb).^51^ Ion straggling is only one of the factors limiting spatial accuracy. The diameter of the impinging ion beam and the diffusion length of the atoms during the thermal annealing step employed to form the vacancy centre complexes play a prime role too (see below).

Arguably the main outstanding challenge to deterministic nanoscale positioning of colour centres in diamond is the availability of specific beam optics for accurate spatial delivery of individual ions. To this end, two main approaches are being pursued to reach resolutions well below 100 nm—nanoscale focusing and scanning of a rarefied ion beam via electromagnetic lenses, and use of collimators in combination with nano-apertures. For the first approach, the main obstacle is the limited focusing capability of conventional MeV energy accelerators.^52^ This has however been overcome by the development—still ongoing—of custom focused ion beam (FIB) apparatuses with commercially available alternatives to ordinary gallium liquid metal ion sources.^53–56^ The second approach has the advantage that achromatic nanoscale collimators are compatible with any ion species as well as energy, and have been used in a variety of configurations to achieve spatially resolved ion implantation. Specifically, collimators can consist of fixed apertures aligned with specific implantation targets on the substrate,^57^ or can be mounted on a scanning system enabling ion delivery at arbitrary positions.^58^

### Single-ion detection

In general, the delivery of a predefined number of ions on the diamond target cannot be achieved by a Poissonian approach, which relies on preset exposure time and ion current.^59^ A much more deterministic solution is needed. Real-time counting of individual ions as they strike the target material is needed to verify that the ion has been implanted. To achieve this, the target substrate itself is engineered to be a solid-state particle detector via patterning of suitable electrode geometries.^60^ This strategy also allows for the exploitation of the same electrodes for subsequent applications, such as inducing Stark shifts and electroluminescence. However, as solid-state single-ion detection relies on the readout of a voltage signal generated through the collection of excess carriers produced by the impact of individual ions on the target, this task is challenged by the high energy required (~13 eV) to generate an electron–hole pair in diamond.^61^ As the height of the induced charge pulse signal generated by an ion impact is proportional to the deposited energy, there is a trade-off between maximizing the signal-to-noise ratio and preserving the desired spatial resolution—the latter being affected by the ion straggling. To date, the detection of Si ions at energies as low as 200 keV has been demonstrated using single-ion counting techniques developed in the context of nuclear microprobe technologies.^60,62^ Note that this detection approach cannot be employed for target implantation in photonic structures, for these cannot intrinsically be fitted with sensing electrodes. In this case, ion counting could be performed through the detection of secondary electrons.^63^ This scheme—yet to be realized for single-ion delivery in diamond devices—would prevent utilizing collimators for nanoscale positioning, as they would absorb the secondary radiation emitted from the target. An alternative path consists in fabricating the photonic structures themselves around a set of emitters pre-implanted at specific positions.^64^ Another emerging approach is the laser writing of colour centres in diamond. This approach also enables the combination of the fabrication process with in situ confocal microscopy and annealing to generate on-demand emitters.^65^

### Formation yield

The incorporation of different ions in diamond is the first step. This is followed by the formation of the implanted impurities into optically active emitters—that is, atom-vacancy complexes. This is achieved by annealing at high temperatures, which promotes the recombination of the ions with implantation-induced lattice vacancies. Typical annealing temperatures are in the 800–1200 °C range for all group IV elements. To date, yields of ≤25%, 4%, 5% and 2% have been reported for 15–50 keV implantation of Si^54^, Ge^55^, Sn^33^ and Pb^35^ ions, respectively. These estimates are statistical, due to the limited amount of experiments exploiting single-ion counting techniques. Promising alternative techniques—including for instance HPHT treatments—might lead to a significant increase in yield. This was demonstrated for other centres in diamond,^66^ as well as for the SnV^–^ with a reported higher stability of the HPHT-treated centres^32^ with respect to those subjected to ordinary annealing.^33^ Another promising approach consists in combining femtosecond laser-induced annealing with conventional thermal treatments.^67^

## Spin-optical properties

Key to demonstrating fundamental phenomena, as well as realizing quantum-based devices, is the ability to address and manipulate the spin state of these diamond colour centres. This aspect is still the object of ongoing research as is the development, in parallel, of protocols to detect, manipulate and convert the charge state of the M-V defects—for these centres can exist in multiple charge states. This degree of control is particularly important for reducing, or ideally eliminating, effects such as spectral diffusion and ionization, which limit the practical employment of these colour centres in quantum applications relying on photon indistinguishability.

### Symmetry and structure

Group IV diamond defects display the split-vacancy configuration in which the element M (Si, Ge, Sn, Pb) lies in between two adjacent diamond vacancies, that is, two missing nearest-neighbour carbon atoms.^13^ The centre has nominally *D*_3d_ symmetry with the diamond <1 1 1> axis as the principal, three-fold rotation axis (*C*_3_)—although lower symmetries (e.g. *C*_2_, *D*_2_ point groups) can occur due to lattice strain and deformation. For completeness, a recent study^68^ reported on the possible existence of another class of silicon-related defects with an inhomogeneous distribution of centre wavelengths (715–835 nm). This suggests that group IV elements in diamond may form optically active complexes beyond the divacancy configuration. In this perspective, we focus on M-V centres in the divacancy configuration as this is the most studied, to date. In the negatively charged state of the defects—which gives the characteristic emission lines in Fig. 1b—there are 11 valence electrons involved: six from the dangling bonds of the adjacent carbon atoms, four from the group IV element and one from elsewhere in the lattice.

The resulting M-V^–^ defects have ground (^2^*E*_g_) and excited (^2^*E*_u_) states that both have *E* symmetry and double orbital degeneracy. The orbital degeneracy is lifted by spin–orbit coupling and dynamic Jahn–Teller interaction, leading to a pair of split ground and excited states, each with double spin degeneracy (*S* = 1/2, Fig. 1c).^14,69,70^ These eigenstates have corresponding optical and phononic transitions, which couple only to the orbital degree of freedom and are spin conserving, allowing for all-optical coherent control schemes of the defect’s spin state. Notably, these spin states possess a near-unity spin purity, enabling spin-tagged resonance fluorescence measurements^18^ and making these colour centres desirable spin–photon quantum interface candidates for quantum information networks. Further, owing to the *D*_3d_ symmetry, the application of a magnetic field of given magnitude and direction determines the admixture of the resultant spin eigenstate and can influence the relative relaxation time as the ground and excited state manifolds experience different strengths of spin–orbit interaction. In the SiV^–^, for instance, tuning the orientation of the magnetic field with respect to the *C*_3_ symmetry axis results in the variation of the spin relaxation time from tens of nanoseconds to a few milliseconds^36^—the longest relaxation time being for the field aligned along *C*_3_.

### Spin control

For many quantum-based applications, the spin coherence of the colour centres is a crucial figure of merit, for it dictates the feasibility as well as the maximum number of fundamental operations executable on the candidate qubit. Ideally, the ratio between the time of a single gate operation and decoherence time of the (spin) state should be ~10^–3^–10^–6^, as imposed by error correction limits. Here we focus on the well-studied SiV^–^ as a representative case; similar considerations though can be drawn for the other diamond M-V centres and are briefly discussed at the end of this section.

For the SiV^–^ the coherence time is relatively short and is thus one of the main challenges towards its use as a robust qubit. The SiV^–^ possesses two possible paths to encode information as a qubit—either via the two orbital branches of the ground state (Δ_GS_ ~47 GHz)^71^ or via the spin *S* = 1/2 sublevels of the orbital states, which have characteristic optical signatures and whose degeneracy is lifted by Zeeman splitting. Compared to the rather long spin relaxation time (*T*_1_^spin^ ~ms), the lifetime of the orbital states of the SiV^–^ centre is limited (*T*_1_^Orbital^ ~tens of ns) due to acoustic phonon scattering between the two ground orbital branches (|12〉 and |34〉 in Fig. 1c).^21,22,72,73^ The dephasing time involving Zeeman-split spin sublevels (*T*_2_^⋆^ ~tens of ns) is also short and of the same order of *T*_1_^Orbital^. It follows the same temperature dependence of *T*_1_^Orbital^, indicating that spin dephasing is also dominated by phonon-mediated transitions between the orbital branches.^22^ For completeness, longer values for the spin dephasing time of the SiV^–^, *T*_2_^⋆^ ~hundred ns, have been reported in Ramsey interferometry experiments measuring the free induction decay (FID) time of the spin.^22^

**Fig. 2:**
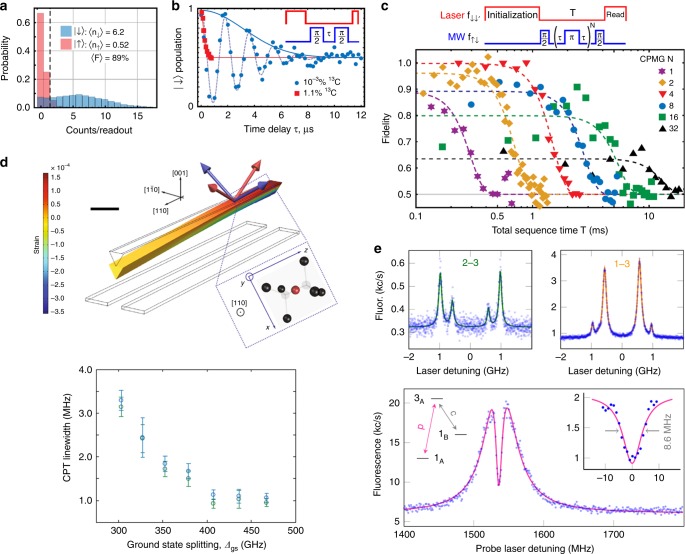
Coherent spin control in the SiV^–^ centre. **a** Spin control of the silicon-vacancy centre at millikelvin temperatures. Single-shot spin readout with magnetic field *B* = 2.7 kG: a 20-ms-long laser pulse pumping the transition A1 (see Fig. 1c) is used to read out the state after a 250-ms-long initialization pump of the A1 (red) or B2 transition (blue). **b** Ramsey interference measurement of *T*_2_^⋆^ for two samples: ^13^C purified sample (blue, 0.001% ^13^C) and unprocessed natural sample (red, 1.1% ^13^C). The microwave field is detuned by ~550 kHz from the Zeeman splitting between |1〉 and |2〉. The duration of the initialization and readout period are 15 and 2 ms for the 0.001% ^13^C sample, and 2 and 1.5 ms for the 1.1% ^13^C sample. **c** Carr–Purcell–Meiboom–Gill (CPMG) pulse sequence with *N* = 1, 2, 4, 8, 16 and 32 pulses in a sample with low ^13^C concentrations and with an aligned magnetic field *B* ≈ 1.6 kG at 100 mK. Durations of the initialization and readout laser pulses are 100 and 15 ms, respectively. Dashed lines are fits to exp[–(*T*/*T*_2_)^4^]. **d** Suppression of spin dephasing via strain engineering. The simulated bending of a diamond cantilever containing a SiV^–^ centre is at an applied voltage of 200 V between top and bottom electrodes. The component of the strain tensor in the direction of the long axis of the cantilever is indicated by the colour scale. Scale bar corresponds to 2 μm. The linewidth of coherent population trapping (CPT) dips as a function of ground-state orbital splitting Δ_GS_ is shown, indicating an increase in spin coherence for higher levels of strain. **e** Demonstration of coherent manipulation trapping with GeV^–^ centres. The top panels represent coherent excitation from the lower (1) and higher (2) energy ground state manifold to the excited state (3). A dip with full-width at half-maximum of (8.6 ± 0.5) MHz is visible, which corresponds to a coherence lifetime of (19 ± 1) ns. Panels a, b and c are reprinted with permission from ref. ^36^. Copyright 2017 by the American Physical Society; panel d is reprinted from ref. ^76^, Copyright 2018 by Springer Nature; panel e is reprinted with permission from ref. ^37^ Copyright 2017 by the American Physical Society.

Being such a critical constraint, different approaches have been proposed to increase the coherence time of the SiV^–^. One option is to cool down the SiV^–^ centre to millikelvin temperatures,^28^ which is currently possible with commercial ^3^He/^4^He dilution refrigerators. At these temperatures, the spin coherence time can reach tens of milliseconds if the magnetic field is well aligned,^36^ as shown in Fig. 2a–c. This allows for optical excitations of the SiV^–^ centre for as many as ~10^5^ cycles before a spin-flip event can occur, thus making high-fidelity single-shot spin readout a concrete possibility, even with low photon collection efficiencies (~10^–4^). At millikelvin temperatures the strong suppression of phonon-mediated dephasing allows for the observation of prominent Rabi oscillations between the two spin ground states. FID measurements have shown that the spin coherence time of the SiV^–^ centre can increase from ~tens or hundreds of nanoseconds for natural samples^74^ to ~10 μs for isotopically pure diamond (0.001% ^13^C). Extension of the spin coherence time is possible by employing dynamical decoupling protocols to rephase the spin via a series of π-pulses. Notably, in these schemes the fact that the spin coherence time does not saturate with the number of π-pulses indicates that the coherence time can be further extended by reducing the uncertainty on the pulses, for instance, by using a decoupling sequence with two-axis control.^75^

A second method to enhance the coherence time of the SiV^–^ centre is to manipulate the phonon scattering strength via strain engineering^76^ (Fig. 2d). Here a tuneable static strain is applied on a SiV^–^ centre that is located in a monolithic single-crystal diamond cantilever with metal electrodes patterned above and below. Voltage applied across the electrodes deflects the cantilever downwards generating a controllable deformation. The strain mixes the orbitals of the ground and excited state manifolds, and alters the splitting (Δ_GS_ and Δ_ES_) in each manifold. Theoretical simulations indicate that, at 4.2 K, increasing the orbital splitting Δ_GS_ would cause the phonon absorption rate of the SiV^–^ centre to first raise slightly (due to the higher phonon density of states), before experiencing a monotonic drop (due to the competing exponential decrease in thermal occupation of the phononic states themselves). Overall, a reduction in phonon absorption causes the suppression of the phonon-mediated dephasing process in the system, effectively increasing the coherence time (Fig. 2d).

## Charge-state control

An indirect alternative is to consider the neutrally charged state of the M-V defects. For instance, the SiV^0^ displays strong (~90%), nearly transform-limited (~450 MHz) emission in the ZPL at 946 nm. It is a *S* = 1 spin system, which is less subject than its negatively charged counterpart to decoherence caused by phonon interaction and electric field fluctuations.^77,78^ Spin polarization of the system—needed to initialize the spin controllably—has been observed,^79^ and explained through state-selective intersystem crossing between singlet and triplet states, similarly to what occurs in NV^–^ centres. Additionally, time-resolved electron spin resonance measurements on ensembles of SiV^0^ centres have shown long coherence times of ~1 ms (with Hahn-echo schemes), as well as exceptionally long spin–lattice relaxation times ~40 s, at temperatures <20 K. The dephasing mechanism seems to be dominated by slowly varying noise from nearby ^13^C nuclear spins, which means it can be reduced by improving the quality and isotopic purity of the material.

The main drawbacks of the SiV^0^ are the relatively low fluorescence at low temperature and unknown precise concentration of boron and nitrogen in the host diamond required to engineer this colour centre efficiently.^80^ So far, it was only identified in specifically grown diamonds, provided by Element Six.^78,80^

Other neutrally charged defects (i.e. PbV^0^, GeV^0^ and SnV^0^) have not been reported yet. This might be due to several facts, including the Fermi level in the diamond lattice being unfavourable to their formation, or their potentially low quantum efficiency preventing detection in standard photoluminescence measurements.

### Spin control of other group IV emitters

Beyond the SiV^–^ centre, spin manipulation using microwave fields has been demonstrated in GeV^–^ centres.^37^ Similarly to the case of the SiV^–^, the spin coherence of the GeV^–^ is limited by phonon-mediated orbital relaxation. This relaxation rate scales quadratically with the spin–orbit splitting in the zero-field limit, and leads to the GeV^–^ having a faster spin dephasing rate than the SiV^–^—due to the GeV^–^ possessing a larger spin–orbit splitting^19^. The spin dephasing time of the GeV^–^ is ~20 ns—as measured through coherent population trapping experiments at cryogenic temperatures (~2.2 K). The results are shown in Fig. 2e. For this measurement, a (1 0 0)-oriented diamond was used with magnetic field applied in the plane of the diamond. The larger-than-usual splitting of the ground state is due to the residual strain.^37^ The GeV^–^ spin relaxation time was measured to be ~25 μs under precise alignment (54°) between the magnetic field and the GeV^–^ axis. The other M-V centres in the family possess larger intrinsic orbital splitting, which suggest that they might be less prone to dephasing. In general, these experimental demonstrations show that the family of diamond M-V centres constitute promising systems for developing spin–photon interfaces and quantum networks communicating via photons.

## Quantum photonics with group IV emitters

Coherent light–matter interfaces are one of the key components of many proposed quantum technologies. They are for instance essential for advanced quantum networks and modular quantum computing architectures^81^ that exploit photon interference to entangle distant long-lived spin-based quantum memories.^82^ In this framework, diamond group IV emitters are suitable candidate hardware. Their strong emission into the spin-correlated ZPL, symmetry-protected optical transitions and low inhomogeneous spectral distribution set them above many alternative systems. Further, nanofabrication in the semiconductor industry is well established, making diamond M-V emitters a concrete route towards realizing integrated nanophotonic devices based on light–matter interactions with single quanta. In this section, we review the use of group IV emitters in some of the main quantum optical technologies.

### Optical properties

**Fig. 3:**
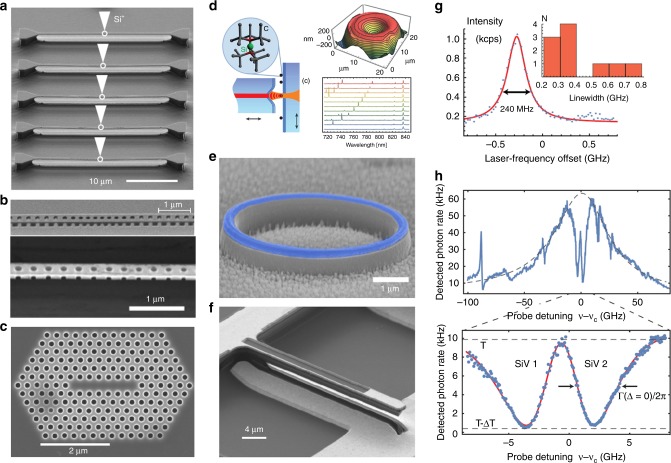
Nanophotonic integration of group IV emitters. Photonic crystals containing group IV diamond colour centres and including: **a** 1D triangular nanobeam cavities (from ref. ^56^. Reprinted with permission from AAAS). **b** Quasi-isotropic etched nanobeams (from ref. ^85^. Reprinted with permission from AAAS). **c** FIB-patterned diamond membranes (figure reproduced with permission from ref. ^86^. Copyright 2012, Springer Nature). **d** Fibre-microcavity integrated SiV^–^ centres (reproduced with permission from ref. ^87^. Copyright 2017 by the American Physical Society). **e** GeV^–^ centres coupled to micro-rings (reproduced with permission from ref. ^88^. American Chemical Society). **f** Group IV strain tuning devices based on electrostatically actuated cantilevers (reprinted with permission from ref. ^89^. Copyright 2018 by the American Physical Society). **g** Optical properties of SiV^–^ centres in 1D photonic structures. The linewidth distribution shows near-lifetime-limited SiV^–^ centres in optical structures (reprinted with permission from ref. ^26^. Copyright 2016 by the American Physical Society). **h** Cavity reflectivity measurements indicating a high cooperativity *C* > 20 (from ref. ^85^. Reprinted with permission from AAAS).

One essential property of a coherent light–matter interface is a transform-limited optical transition. This is because in the opposite case, that is, when spectral broadening is present, the emitted photons are distinguishable and the fidelity of key linear-optical quantum operations (e.g. projective Bell-state measurements) is limited. A second desirable property is high quantum efficiency: emission of spectrally coherent, spin-correlated photons into a specific mode with high probability after optical excitation. Finally, the inhomogeneous (emitter-to-emitter) variation in optical transition frequency should be small such that spectrally identical emitters can be produced scalably. Group IV defects in diamond have advantages over other solid-state emitters for these crucial aspects. Unlike NV^–^ centres, which have poor optical linewidths when produced through ion implantation,^83^ SiV^–^ centres have been shown to have highly coherent optical transitions when incorporated in this fashion. A similarly high photon coherence has since been demonstrated for GeV^–^ and SnV^–^, showing that the inversion symmetry of the diamond M-V defects makes them robust in this respect. As mentioned before, diamond group IV emitters generally have higher Debye–Waller factors than NV^–^ centres, resulting in higher emission efficiency into the ZPL and can have very small inhomogeneous spectral distribution. As a result, in 2014 the experimental demonstration of Hong–Ou–Mandel interference between two SiV^–^ centres was realized.^27^ The experiment set a milestone for diamond-based quantum optics and proved that group IV emitters are serious contenders for advanced solid-state quantum realizations.^84^ The key properties of group IV emitters in this context are summarized in Fig. 3.

### Device integration

To further increase the quality of the light–matter interface, the emitters can be integrated into photonic devices.^90,91^ By structuring the electromagnetic environment surrounding the emitter, light–matter interaction can be increased via the cavity-quantum electrodynamics cooperativity *C* = 4*g*^2^/*kγ*, where *g* is the emitter–photon coupling rate, *k* the loss rate of the optical mode and *γ* the emitter spontaneous emission rate. The cooperativity is related to several important measures. One is the Purcell factor *F*_P_ = *C*/*QE*, with (1p – *QE*) quantifying the emission into all non-cavity modes, which increases the emission rate and reduces the emitter lifetime, thus reducing the effect of inhomogeneous spectral broadening. A second quantity is the *β*-factor, which quantifies the emission efficiency into a single mode and scales proportionally to *C*/(1 + *C*). The cooperativity also defines cavity transmission and reflectivity in the presence of an emitter, key measures of quantum operation fidelities in networking architectures—for example, the Duan–Kimble scheme.^92^ Diamond nanophotonic cavities with high-quality factors, *Q* > 10,000, and with low mode volume, *V* ~ (*λ*/2*n*)^3^, have been demonstrated in several realizations.^6^ However, the high optical dephasing rate of NV^–^ centres—for which the cavities were designed—near-etched surfaces, combined with the high emission of the NV^–^ into the phonon sideband, limited achievable cooperativity to *C* < 1 in these systems.

Nanophotonic integration of group IV emitters has since overcome these issues. The first demonstration of coupling of group IV emitters to photonic crystal cavities in bulk diamond dates back to 2012.^86,93^ The SiV^–^ centres were integrated in one- (1D) and two-dimesional FIB-etched structures limited by a relatively low *Q* of 700 or to a microdisk cavity. In the following years, improved fabrication techniques based on angular^94^ and quasi-isotropic^95,96^ oxygen etching led to increased photonic crystal cavity quality factors, resulting in significant advances towards strong light–matter interaction (Fig. 3a–c). Other photonic configurations have also been explored, including fibre-based microcavities,^87,97^ where Purcell factors of 9.2 have been shown, and diamond microdisk resonators^88^ (Fig. 3d, e), and nanopillars.^34,98,99^ Hybrid approaches coupling SiV centres to SiC photonic structures such as nanopillars^100^ and microdisk resonators^101^ have also resulted in emission enhancement.

At the same time, the use of group IV emitters has reduced spectral broadening, with near-lifetime-limited linewidths reported for SiV^–^ centres in single-mode diamond waveguides^26^ (Fig. 3g). This combination of improved nanofabrication and reduced spectral width enabled the recent demonstration of high-cooperativity (*C* ~ 20) light–matter interfaces using SiV^–^ centres coupled to 1D photonic crystals,^85^ such that high-fidelity gates between single spins and travelling photons^92^ are now possible (Fig. 3h). Finally, the inhomogeneous spectral distribution of emitters has been overcome through the use of strain tuning, where electromechanical cantilevers are employed to bring multiple group IV centres into spectral alignment^76,102^ (Fig. 3f). With the addition of long spin coherence and high-fidelity control described above, nanophotonic-integrated group IV emitters have all the properties required for coherent light–matter interface applications.

**Fig. 4:**
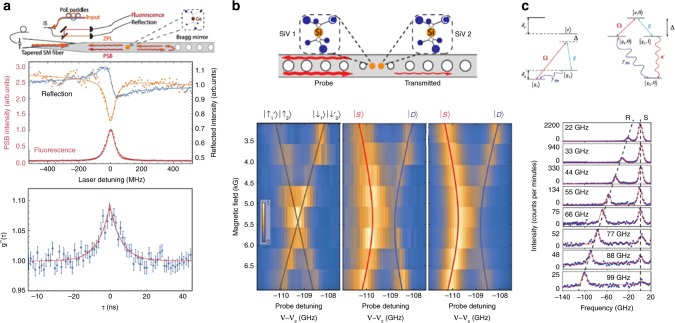
Quantum optics with group IV colour centres. **a** Homodyne interferometry with a single GeV centre (top panel).  Interference between GeV resonance fluorescence and near-resonant excitation laser light reflected in the fibre by the Bragg mirror. Varying their relative amplitude and phase, by modifying the polarization of the input laser, results in the change in line shape of the output light (mid panel) from symmetric, corresponding to destructive interference (orange) to dispersive (blue). The Hanbury Brown-Twiss interferometry measurement (bottom panel) shows *g*^2^(0) > 1 due to interference between the excitation laser and the resonant fluorescence from the single GeV centre. It highlights the quantum nonlinear character of the coupled GeV-waveguide system (reprinted with permission from ref. ^103^. Copyright 2017 by the American Physical Society). **b** Coherent coupling between two SiV^–^ centres in a photonic crystal cavity. The characteristic avoided crossing (mid panel) indicates a coupling greater than the optical linewidth (from ref. ^85^. Reprinted with permission from AAAS). **c** Widely tuneable Raman emission from a SiV^–^ centre coupled to a photonic crystal cavity (reprinted with permission from ref. ^104^. Copyright 2018 by the American Physical Society).

These exceptional properties have been employed for several advances in quantum optics. For instance, SiV^–^ and GeV^–^ centres in nanophotonic waveguides have been used in demonstrations of superradiant emission, indicating entanglement between spatially separate emitters in a single optical mode (Fig. 4a).^56,103^ This set group IV diamond emitters as candidate fundamental building blocks for quantum networks relying on distant entanglement of quantum nodes. Further work extended this concept to two emitters mutually coupled to a cavity mode.^85^ With the increased light–matter coupling afforded by the cavity, coherent interactions can be directly observed via an avoided crossing in the optical spectra as the emitters are magnetically tuned onto resonance with each other (Fig. 4b). Strong, controllable coupling as demonstrated in this work can enable deterministic two-qubit gates, an essential element of a quantum repeater node. A third advance is the production of spectrally tuneable coherent single photons via cavity-assisted Raman processes^104^ (Fig. 4c). This scheme has the potential for producing indistinguishable single photons in a scalable manner, which is the core requirement for linear-optical quantum computing and network applications. The robust optical coherence and lack of spectral diffusion in group IV emitters integrated with nanophotonics, including lifetime-limited SiV^–^ and SnV^–^, as well as spectrally narrow GeV^–^, are some of the key elements that enabled these breakthrough demonstrations of light–matter interaction. Further, the demonstrated coherent spin control with nanophotonic-integrated group IV systems, including ancilla nuclear spin ‘data’ qubits,^105^ sets the concrete possibility for the realization of a fully-functional multi-qubit quantum network node.

## Quantum plasmonics

**Fig. 5:**
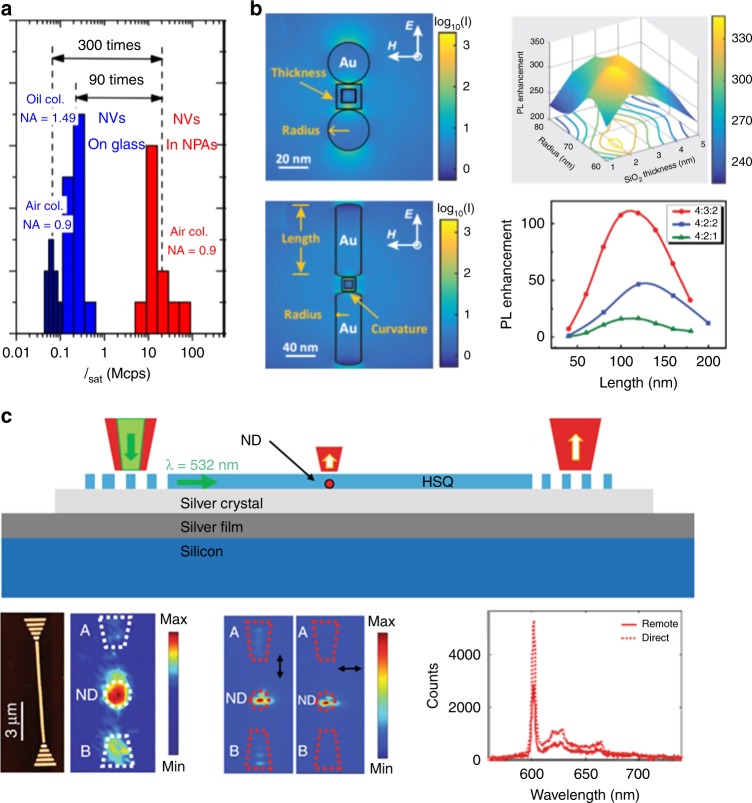
Integrated plasmonics with group IV defects in diamond. **a** State-of-the-art enhancement of luminescence from a single NV^–^ centre, resulting in over 1 × 10^6^ counts/s at saturation (reproduced and adapted with permission from ref. ^108^. Copyright 2018 American Chemical Society). **b** Modelling of SiV^–^ photoluminescence enhancement in an antenna configuration, showing a potential 300-fold enhancement (reproduced and adapted with permission from ref. ^109^. Copyright 2017, by the American Physical Society). **c** Example of a simple plasmonic waveguide coupled to GeV^–^ centres. Coupling to the waveguide is demonstrated, and remote collection through the waveguide is shown to be better than a direct one (reproduced and adapted from ref. ^110^, Copyright 2018, by Springer Nature).

Colour centres in diamond have always been one of the most prominent systems for quantum plasmonic experiments owing to their robustness and small size.^106^ For instance, NV^–^ centres in nanodiamond have led to the demonstration of wave-particle duality of surface plasmons,^107^ and have been successfully coupled to a variety of resonators and waveguides. Remarkably, in a recent experiment a single nanodiamond was positioned in an ultra-small gap cavity and resulted in a two-order-of-magnitude enhancement in luminescence from a hosted single NV^–^ centre, achieving >10 Mcounts/s at saturation at room temperature^108^ (Fig. 5a).

Beyond these first realizations exploiting the well-characterized NV^–^ centre, the emergence of the group IV emitters in diamond—with their narrowband, fully-polarized emission—brought a new wave of experiments as well as novel plasmonic structure designs. Recently, a specific geometry involving gold dimers has been proposed to achieve a three-hundred-fold luminescence enhancement from SiV^–^ colour centres in a diamond nanoparticle.^109^ Interestingly, the proposed dimensions and geometry are within technological reach as they require a 10-nm nanodiamond coated with few-nm SiO_2_ film and located between two gold structures ~65–120 nm in size, depending on the geometry of the dimer (Fig. 5b). The experimental demonstration of this particular proposal is yet to be achieved; nevertheless, the first steps towards on-chip quantum plasmonics with group IV emitters have already been taken. Figure 5c shows a GeV^–^ centre in a nanodiamond host coupled to a hybrid plasmonic waveguide.^110^ The nanodiamond containing a single GeV^–^ centre is embedded in a hydrogen silsesquioxane waveguide on top of a smooth silver film. The photoluminescence signal from the GeV^–^ shows a modest three-fold enhancement, and, more importantly, the same scheme allows for excitation of the GeV^–^ remotely, through the grating coupler. Although the waveguide and cavity lengths in this particular example are small (~tens of μm), longer lengths are technologically feasible with the use of more suitable dielectric crystals (e.g. TiO_2_) and larger single-crystal silver films.

New designs and calculations are also being brought forward for realizing indistinguishable photon sources at room temperature based on group IV colour centres in diamond.^111^ The basic idea revolves around employing low-*Q* cavities that have ultra-small volume and can lead to strong light confinement and fluorescence enhancement. This approach has already been realized experimentally to demonstrate ultra-bright emission (yet not of coherent or indistinguishable photons) in other systems, including NV^–^ centres, emitters in 2D materials,^112^ and quantum dots,^113^ with count rates ~MHz. Current losses in the plasmonic waveguides and devices can be minimized by synthesis and growth of better-quality materials, by careful geometric design and improved coupling to the far field. The latter can be achieved, for instance, via a nanoparticle acting both as a scatterer and, simultaneously, as an antenna. If the spontaneous emission is decreased to ultimately reach the dephasing limit, experiments in the quantum regime could be performed at liquid nitrogen and perhaps even at room temperature.^111^

## Applications, outlook and challenges

**Fig. 6:**
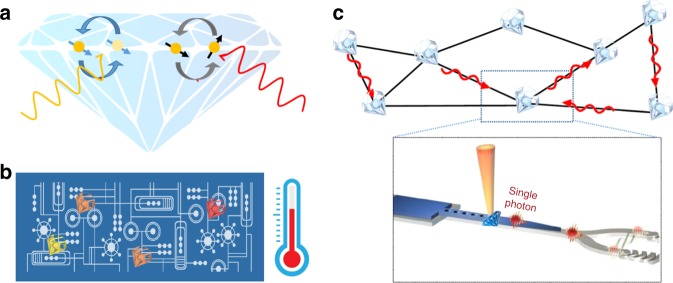
Schematic of some of the envisioned applications and demonstrations for group IV emitters in diamond. **a** All-optical charge- and spin-state control of group IV emitters in diamond—whereby a laser pulse can change the charge state of the emitter, or coherently manipulate its spin state. **b** Quantum sensing, for example, nanothermometry of integrated circuits, using M-V defects in diamond nanoparticle hosts. The nanodiamonds can be positioned onto the circuit and used to measure the temperature with high sensitivity and spatial resolution by monitoring their photoluminescence. **c** Quantum network based on diamond group IV emitters as spin–photon interfaces. The system consists of a diamond photonic crystal with a tapered edge coupled to a waveguide made from, for example, aluminium nitride (adapted from refs. ^120,121^).

Group IV diamond emitters have already been successfully employed in a suite of fundamental demonstrations and practical applications. Yet, the field is still at its infancy, especially in the context of controlling certain fundamental properties of the emitters (e.g. quantum efficiency) and exploring specific research areas (e.g. sensing). An in-depth discussion of these is beyond the scope of this perspective, but we deem important highlighting a few notable ones where M-V emitters are showing great promise (Fig. 6).

Quantum sensing has been a particularly active area of research for diamond M-V centres. Atom-like in nature, encased in a robust diamond matrix and mere nanometres away from the surface, these colour centres are ideal sensing elements. These features have been recently explored to measure, for instance, temperatures at the nanoscale via all-optical approaches. The methods rely on monitoring the ZPL amplitude, barycentre or full-width at half-maximum of the emitters as the temperature varies^114–116^ or, in more sensitive realizations, on detecting changes in their fluorescence intensity under anti-stokes excitation—which probes the highly temperature-dependent phonons.^117^ Given that SiV^–^ centres can be effectively integrated into atomic force microscopy tips,^118^ such a method can be a strong contender for all-optical scanning thermometry.

Additional applications that warrant attention in the context of sensing are labelling and imaging in liquid, particularly with diamond nanoparticles in biological environments. Here, diamond group IV defects have significant advantages over alternative biomarkers. The diamond particles can be as small as just a few nanometers,^119^ they are non-toxic and can be readily functionalized to be target-specific. The hosted M-V emitters have narrowband emission at different wavelengths, which allows for multi-wavelength imaging—nanodiamonds containing different colour centres can be functionalized to target different biological structures and monitored simultaneously through different optical filters. An aspect that is particularly appealing from a practical point of view is in situ-doping during HPHT growth, which can produce large quantities (~kg) of nanodiamonds. This can be achieved via addition of silicon- or germanium-based precursors, which are readily available.

Group IV emitters in diamond are also showing potential for electroluminescence-based applications where the luminescence of the defect is triggered electrically rather than optically. Unlike NV^–^ centres, SiV^–^ centres can be driven electrically.^122–125^ It has been proposed that other group IV defects could also be excited electrically. This area of research is still at an early stage, but the preliminary results and the forecast predictions are promising. Diamond diodes have been already demonstrated, although on-set voltages and currents are quite high, mostly due to challenges in achieving good quality n-type doping. A promising pathway forward is a hybrid approach, involving for instance p-type diamond and an external n-type material (e.g. indium tin oxide) to realize an optical diode.^123^ Such control can be exploited to fabricate scalable, on-chip quantum circuitry—ideally comparable to state-of-the-art GaAs-based quantum dots—as well as to achieve tuning of spin transitions and generation of coherent photons from the colour centres. This is particularly advantageous for the long-term vision of realizing full integration on a single chip, where optical excitation is limited by the diffraction limit.

Another direction where more research is needed is charge-state control. While the negatively charged M-V^–^ emitters in diamond are well studied, so far only the neutrally charged SiV^0^ centre has been experimentally observed and characterized. The SiV^0^ is promising, as it offers a combination of long electron spin coherence and desirable optical properties—strong (~90%), nearly transform-limited (~450 MHz) emission in the ZPL in the near infrared at 946 nm. However, engineering the neutrally charged variant of M-V defects on demand has, so far, proven challenging as the corresponding negatively charged defects seem to form more favourably. Current strategies rely on doping the diamond with boron atoms (which can act as acceptors) while limiting the amount of nitrogen atoms (which can act as donors). The precise conditions to achieve a deterministic yield are still under study. There is also an ongoing debate about the feasibility of isolating single SiV^0^ defects in a controllable manner.

Overall group IV diamond emitters combine desirable optical properties—comparable to quantum dots—and long spin coherence time—similar to NV^–^ centres. We thus also anticipate a bloom of advanced quantum applications requiring spin–photon interfaces. One of the most ambitious realizations consists in the design of a prototype quantum network based on spin–photon and spin–spin entanglement mediated by photon interference.^2^ Research on strong coupling between group IV emitters and cavities, as well as the implementation of spin–photon controlled phase gates mediated by the cavity, is currently ongoing, as is the investigation of spin–spin entanglement via direct dipole–dipole coupling and the realization of a qubit platform where nuclear spins are employed as quantum memories.
