# Author Correction: Quantum nanophotonics with group IV defects in diamond

**DOI:** 10.1038/s41467-020-14316-x

**Published:** 2020-01-14

**Authors:** Carlo Bradac, Weibo Gao, Jacopo Forneris, Matthew E. Trusheim, Igor Aharonovich

**Affiliations:** 1 School of Mathematical and Physical Sciences, Faculty of Science, University of Technology, Sydney, NSW 2007 Australia; 2 Division of Physics and Applied Physics, School of Physical and Mathematical Sciences, Nanyang Technological University, Singapore, 637371 Singapore; 3 Istituto Nazionale di Fisica Nucleare (INFN) and Physics Department, Università degli Studi di Torino, Torino, 10125 Italy; 4 Department of Electrical Engineering and Computer Science, Massachusetts Institute of Technology, Cambridge, MA 02139 USA

**Keywords:** Nanoscience and technology, Optics and photonics

Correction to: *Nature Communications* 10.1038/s41467-019-13332-w, published online 09 December 2019.

The original version of this Article contained an error in the second and third sentences of the legend of Fig. 4, which incorrectly read ‘Superradiant emission of GeV^−^ centres coupled to a single-mode waveguide. The bunching signature with *g*^2^ > 1 (lower left) indicates cooperative emission between two separated GeV^−^ centres’. The correct version states ‘Homodyne interferometry with a single GeV centre (top panel). Interference between GeV resonance fluorescence and near-resonant excitation laser light reflected in the fibre by the Bragg mirror. Varying their relative amplitude and phase, by modifying the polarization of the input laser, results in the change in line shape of the output light (mid panel) from symmetric, corresponding to destructive interference (orange) to dispersive (blue). The Hanbury Brown-Twiss interferometry measurement (bottom panel) shows *g*^2^(0) > 1 due to interference between the excitation laser and the resonant fluorescence from the single GeV centre. It highlights the quantum nonlinear character of the coupled GeV-waveguide system’. This has been corrected in both the PDF and HTML versions of the Article.

